# Add-on PD-1 inhibitor with Peg-IFNα therapy favors functional cure of chronic hepatitis B patients

**DOI:** 10.1515/jtim-2026-0018

**Published:** 2026-02-13

**Authors:** Yaping Li, Chenrui Liu, Liu Yang, Ling He, Mei Li, Guoe Gou, Dandan Feng, Xin Zhang, Fusheng Wang, Junliang Fu, Shuangsuo Dang

**Affiliations:** Department of Infectious Diseases, The Second Affiliated Hospital of Xi'an Jiaotong University, Xi'an, Shaanxi Province, China; Senior Department of Infectious Diseases, Chinese PLA General Hospital, National Clinical Research Center for Infectious Diseases, Beijing, China

**Keywords:** hepatitis B virus, chronic hepatitis B, PD-1 inhibitor, sintilimab, pegylated interferon-α, HBsAg clearance, functional cure

## Abstract

**Background and Objectives:**

The treatment options for hepatitis B surface antigen (HBsAg) clearance are limited for patients who have failed pegylated interferon-α (Peg-IFNα) therapy. Given preclinical evidence that PD-1 blockade boosts antiviral immunity, this study aims to evaluate the efficacy and safety of PD-1 inhibitors in chronic hepatitis B (CHB) patients who have failed interferon therapy.

**Methods:**

This non-randomized controlled trial was conducted in CHB patients who failed to achieve HBsAg clearance after 48 weeks of Peg-IFNα therapy. Including two groups: (i) a PD-1 inhibitor plus Peg-IFNα group receiving sintilimab 1 mg/kg/12 weeks plus Peg-IFNα 180 μg/week (*n* = 33) or (ii) a Peg-IFNα group continuing Peg-IFNα therapy (*n* = 31). The primary endpoint was HBsAg clearance at week 24.

**Results:**

The PD-1 inhibitor plus Peg-IFNα group included 33 patients and the Peg-IFNα group included 31 patients. At week 24, HBsAg clearance occurred in 30.3% of PD-1 inhibitor plus Peg-IFNα group *versus* 9.7% of Peg-IFNα group (*P* = 0.047). Median HBsAg declined by –0.916 log_10_ IU/mL in the PD-1 inhibitor plus Peg-IFNα group compared with –0.067 log_10_ IU/mL in Peg-IFNα group (*P* = 0.013). In the PD-1 inhibitor plus Peg-IFNα group, alanine transaminase (ALT) rose transiently at week 12 only among patients who ultimately cleared HBsAg. Sintilimab increased stomatitis (12.1%) and recurrent fever (36.4%), but the frequency of grade ≥ 3 adverse events did not differ from Peg-IFNα group. Enzyme-linked immunosorbent assay (ELISA) profiling showed progressive induction of ISG15 after sintilimab, and patients achieving HBsAg clearance displayed higher baseline and week-12 OAS1 concentrations than non-clearers (both *P* < 0.05).

**Conclusions:**

Quarterly sintilimab add-on therapy triples the short-term HBsAg clearance rate in interferon-refractory CHB without major additional toxicity. A week-12 ALT surge and elevated OAS1 may serve as early biomarkers of response, but confirmation in larger randomized trials is needed.

## Introduction

Functional cure of chronic hepatitis B (CHB) is defined as sustained clearance of hepatitis B surface antigen (HBsAg) and suppression of hepatitis B virus (HBV) DNA, with or without seroconversion to hepatitis B surface antibody.^[1]^ Achieving this outcome can prevent disease progression to liver cirrhosis and hepatocellular carcinoma (HCC).^[2,3]^ China has the highest HBV disease burden worldwide, with approximately 75 million chronically infected individuals,^[4,5]^ and HBV accounting for 86% of primary HCC cases.^[6]^ Current antiviral therapies include nucleos (t) ide analogues (NAs) and interferons. Pegylated interferon-α (Peg-IFNα), as an immune modulator, plays a key role in CHB treatment.^[7]^

The combination of Peg-IFNα and NAs is a fundamental strategy in the treatment of CHB, aiming for functional cure. Peg-IFNα exerts antiviral effects primarily through activation of the type I interferon signaling pathway. However, the clinical efficacy remains limited, as only a minority of patients achieve HBsAg clearance after 48 weeks of treatment. This highlights the need for novel therapeutic approaches to enhance functional cure rates. Recent advances in CHB immunopathogenesis have drawn attention to immune checkpoint pathways, especially PD-1/PD-L1, which suppress T cell activation and contribute to immune exhaustion. PD-1 is a regulatory receptor on T cells that limits immune activation.^[8,9]^ Chronic HBV infection leads to persistent antigen exposure, upregulating PD-1 and promoting T cell exhaustion.^[10,11]^ Blocking PD-1 signaling may restore antiviral T cell function and enhance HBV clearance.^[12]^ Emerging studies suggest that combining PD-1 inhibitors with Peg-IFNα may improve outcomes in CHB.^[12,13]^ Studies have shown that interferons can induce PD-1 expression on CD8^+^ T cells in CHB patients,^[14,15]^ potentially dampening therapeutic responses. PD-1 inhibition may overcome this immune suppression and reinvigorate antiviral immunity. We also observed in our clinical work that a considerable proportion of CHB patients receiving Peg-IFN treatment presented elevated levels of PD-1 in their peripheral blood T cells. Our preliminary results from a small controlled study showed that adding a PD-1 inhibitor to Peg-IFNα reinitiated HBsAg decline in some patients and recapitulated interferon-related immune activity. These findings, along with reports of HBsAg clearance in patients with HCC receiving PD-1 inhibitors, prompted this trial.

This study aims to evaluate the efficacy and safety of combining PD-1 blockade with Peg-IFNα in CHB patients who failed to achieve HBsAg clearance after 48 weeks of Peg-IFNα therapy. We hope that this study can provide new treatment ideas for CHB patients, especially those who do not respond well to conventional treatment and are in search of more effective functional cure options. We also explore immune mechanisms underlying treatment response using enzyme-linked immunosorbent assay (ELISA)-based cytokine profiling to support the development of precision immunotherapeutic approaches.

## Patients and methods

### Patient inclusion criteria

All patients voluntarily participated in this study and provided written informed consent. The inclusion criteria for patients with CHB were as follows: age 18–65 years; HBsAg positivity for more than 6 months; treated with NAs for more than 1 year prior to the combination of NAs with Peg-IFNα therapy and achieved HBsAg levels < 1500 IU/mL, HBeAg negativity and biological inhibition (defined as high-sensitivity HBV DNA test results of < 20 IU/mL); failure to achieve HBsAg clearance after 48 weeks of Peg-IFNα therapy; and an elevated PD-1 level in peripheral blood T cells (evidenced by an increase compared with the upper limit of the reference range established in our clinical laboratory’s standard test report in one or more subsets of T cells: CD3^+^PD-1^+^ > 40.82%, CD4^+^PD-1^+^ > 42.10%, or CD8^+^PD-1^+^ > 41.00%, as determined by flow cytometry).

### Patient exclusion criteria

The following patients were excluded from this study: those with other conditions that have a poor prognosis, such as human immunodeficiency virus (HIV) infection or malignant tumors; individuals with a history of allergies to biological agents and sintilimab; patients with autoimmune diseases; individuals with positive autoantibodies or hematological disorders; patients treated with drugs that significantly affect the immune system, such as thymosin, immunosuppressants, or glucocorticoids; those with contraindications for interferon therapy or who could not continue interferon treatment; patients with evidence of acute severe liver injury, defined as alanine transaminase (ALT) ≥ 10 times the upper limit of normal (ULN); pregnant or breastfeeding individuals; those with a clear history of autoimmune diseases; individuals with a history of severe psychiatric disorders; patients with a history of severe seizures or who had been treated with anticonvulsants; individuals with thyroid diseases not well controlled after standardized treatment; those with a medical history of severe retinopathy or clinically relevant ophthalmic diseases; patients participating in other clinical trials; individuals unable or unwilling to provide informed consent or adhere to study requirements; patients with a history of allergies to NAs or interferons; and patients who met any contraindications listed in the package inserts for the medications used in this study.

### Enrollment and study design

**Figure 1:**
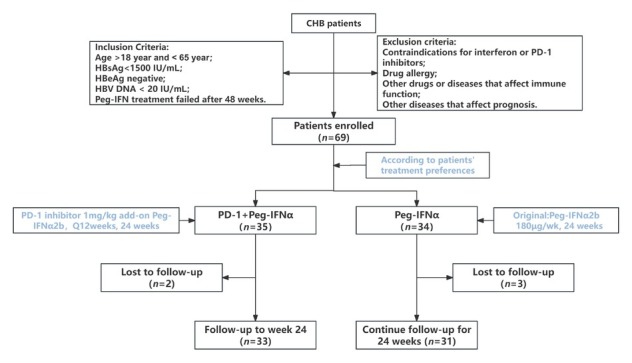
Study design diagram. CHB: chronic hepatitis B; Peg-IFNα: pegylated interferon-α.

This single-center, nonrandomized controlled trial (ChiCTR2400091948) enrolled patients with CHB who failed to achieve HBsAg clearance after 48 weeks of Peg-IFNα therapy. This study was conducted in strict accordance with the Declaration of Helsinki and was approved by the Medical Ethics Committee of Xi’an Jiaotong University Second Affiliated Hospital (Approval No. [2024] Ethic Review-Research No. 028). Written informed consent was obtained from all participants, who were fully informed of the study objectives, procedures, potential risks, and benefits. During the study, patient personal information and clinical data were anonymized and managed in accordance with relevant data protection regulations to ensure the privacy and confidentiality of patient information. Patients were allocated into two groups based on treatment preference: a PD-1 inhibitor plus Peg-IFNα group receiving sintilimab (1 mg/ kg/12 weeks, two doses total) combined with Peg-IFNα (180 μg/week) and NAs, or a Peg-IFNα group continuing their original Peg-IFNα regimen plus NAs. Previous studies have shown that sintilimab maintains 95% receptor occupancy even at a dose of 1 mg/kg, so the dose of sintilimab in this study was selected as 1 mg/kg.^[16]^ The primary endpoints were HBsAg clearance rate at 24 weeks and safety. The overall study design is illustrated in Figure 1.

### Clinical efficacy and safety assessments

During treatment, the HBsAg level, white blood cell (WBC) count, red blood cell (RBC) count, hemoglobin (HB) level, platelet (PLT) count, ALT level, and aspartate transaminase (AST) level of patients were measured at baseline and weeks 4, 12, and 24. HBsAg was quantified using an Architect assay (Abbott Diagnostics; detection limit 0.05 IU/mL), while HBV DNA was measured by real-time PCR. Patients were monitored onsite or *via* phone calls on days 1, 4, 12, and 24 post-PD-1 inhibitor administration. All adverse events (AEs), particularly immune-related liver injury or other immune-mediated reactions associated with PD-1 inhibitors and Peg-IFNα, were carefully documented.

### Flow cytometric analysis

**Figure 2:**
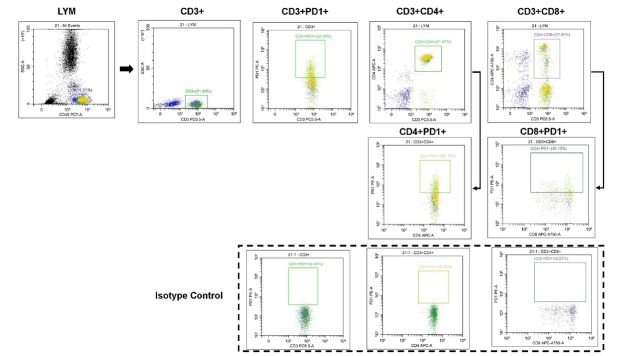
Gating strategy for the detection of PD-1 in the peripheral blood by flow cytometry. After gating the lymphocyte subset from whole blood using a polygon gate, CD3^+^ T cells were identified within this population. Subsequently, rectangular gates were applied within the CD3^+^ T cells to distinguish CD3^+^CD4^+^ T cells and CD3^+^CD8^+^ T cells, followed by further stratification into CD3^+^PD-1^+^ T and CD3^+^PD-1^+^ T subsets. Finally, within the CD3^+^CD4^+^ T cell and CD3^+^CD8^+^ T cell populations, rectangular gating was used to define CD4^+^PD-1^+^ T / CD4^+^PD-1^+^ T cells and CD8^+^PD-1^+^ T/CD8^+^PD-1^+^ T cells, respectively.

Baseline peripheral blood samples from all 64 patients were collected using ethylenediaminetetraacetic acid (EDTA) anticoagulant tubes for flow cytometry to evaluate their PD-1 levels in the peripheral blood. Samples underwent flow cytometric analysis with PD-1 detection kits (Qingdao Raisecare Biotechnology). After collection, 5 μL of CD3^-^ PC5.5, CD4^-^APC-A, or CD8^-^APC-A750-A was added to each flow cytometry tube. In the positive control tube, 5 μL of PD 1-PE-A was included, and 5 μL of the IgG-PE-A isotype control antibody for PD1 was added to the negative control tube. The samples were mixed thoroughly and incubated in the dark for 20 min, followed by the addition of 1 mL of lysis buffer. The samples were allowed to stand for 10 min before flow cytometry analysis. Flow cytometry was performed using the Beckman platform (CytoFLEX, Beckman Coulter, Inc., USA), and Cytexpert software was used for analysis of the results. The gating strategy is shown in Figure 2. In the flow cytometry experiment, the threshold for the FSC channel was set to “ > 50,000 events”, while thresholds for all other channels were set to “ > 10,000 events”. The gain values were configured as follows: FSC: 60; SSC: 100; PE: 80; FITC: 80; PC5.5: 170; APC-A: 200; APC-A750: 200.

### Cytokine measurement by ELISA

Peripheral blood samples were collected from patients in both groups at baseline and weeks 12 and 24 for the PD-1 inhibitor plus Peg-IFNα group, and at baseline and week 24 for the Peg-IFNα group. Serum concentrations of 9 type I interferon pathway-related cytokine (IFIT1, MX1, IFI6, ISG15, OAS1, EIF2AK2, STAT1, IRF7, IFITM3) were measured using commercial ELISA kits (Sino Best Biological Technology, Shanghai). The sample was diluted 5 times during the measurement, and the concentration gradient of the standard dilution for each cytokine measurement is shown in Supplementary Table S1.

### Sample size estimation

We initially intended to conduct a small-sample exploratory study, and accordingly set the significance level (α) at 0.1. Sample size calculation was performed using G Power 3.1.9.7 with parameters set for a two independent samples 𝒵 test, assuming anticipated HBsAg clearance rates of 30% in the PD-1 inhibitor treatment group and 9% in the Peg-IFNα treatment group. With α set at 0.1, power (1-β) at 0.8, and an allocation ratio of 1:1, the required sample size was estimated to be 32 participants per group, yielding a total of 64 participants.

### Statistical analysis

Statistical analyses of 64 patients were conducted using SPSS (version 26.0, Chicago, IL, USA). Continuous variables were summarized as mean ± SD or median (range). Comparisons were performed using Student’s *t*-test, Mann-Whitney *U* test, or repeated-measures analysis of variance (ANOVA). Categorical variables were analyzed as counts and percentages, and comparisons were performed using χ^2^ or Fisher’s exact test. Kaplan-Meier methods evaluated HBsAg clearance rates, with intergroup comparisons conducted by log-rank tests. HBsAg dynamic changes were log-transformed (log [0.025] used for values below detection). All the statistical tests were two-sided, and *P* < 0.05 was considered statistically significant.

## Results

### Demographic characteristics

A total of 69 patients were screened, of whom five withdrew before receiving study medication (three in the PD-1 inhibitor plus Peg-IFNα group and two in the Peg-IFNα group). Reasons for withdrawal were scheduling conflicts (*n* = 2) and family obligations (*n* = 3); none were related to efficacy concerns or AEs. Sixty-four patients were ultimately enrolled: 33 in the PD-1 inhibitor plus Peg-IFNα group and 31 in the Peg-IFNα group. Baseline HBsAg concentrations were comparably low in both cohorts (PD-1 inhibitor plus Peg-IFNα group: 8.90 (2.01–84.07) *vs*. Peg-IFNα group: 2.62 (1.14–78.00) IU/mL, *P* = 0.289). Complete demographic details appear in Table 1.

**Table 1:** Demographic and baseline characteristics of enrolled patients with CHB on antiviral treatment

Characteristics	PD-1 inhibitor plus Peg-IFNα group (*n* = 33)	Peg-IFNα group (*n* = 31)	*P* value
Average age, years	42.79±10.13	45.78±10.51	0.174
Male, *n* (%)	21 (63.6)	21 (67.7)	0.730
Median HBsAg, IU/mL (range)	8.90 (2.01-84.07)	2.62 (1.14-78.00)	0.289
Median ALT, U/L (range)	31.00 (24.00-38.00)	35.00 (26.00-42.00)	0.577
Average CD3PD-1+T cell (%)	45.67 ± 9.41	45.19 ± 9.21	0.839
Average CD4PD-1+T cell (%)	52.73 ± 8.57	50.48 ± 9.91	0.337
Average CD8PD-1+T cell (%)	42.34 ±13.25	44.23 ± 13.30	0.574
Oral HBV therapy, *n* (%)			0.910
Entecavir	11 (33.3)	9 (20.0)	
Tenofovir disoproxil Fumarate	12 (36.4)	10 (32.3)	
Tenofovir alafenamide fumarate	10 (30.3)	12 (38.7)	

CHB: chronic hepatitis B; Peg-IFNα: pegylated interferon-α.

### HBsAg clearance

**Figure 3:**
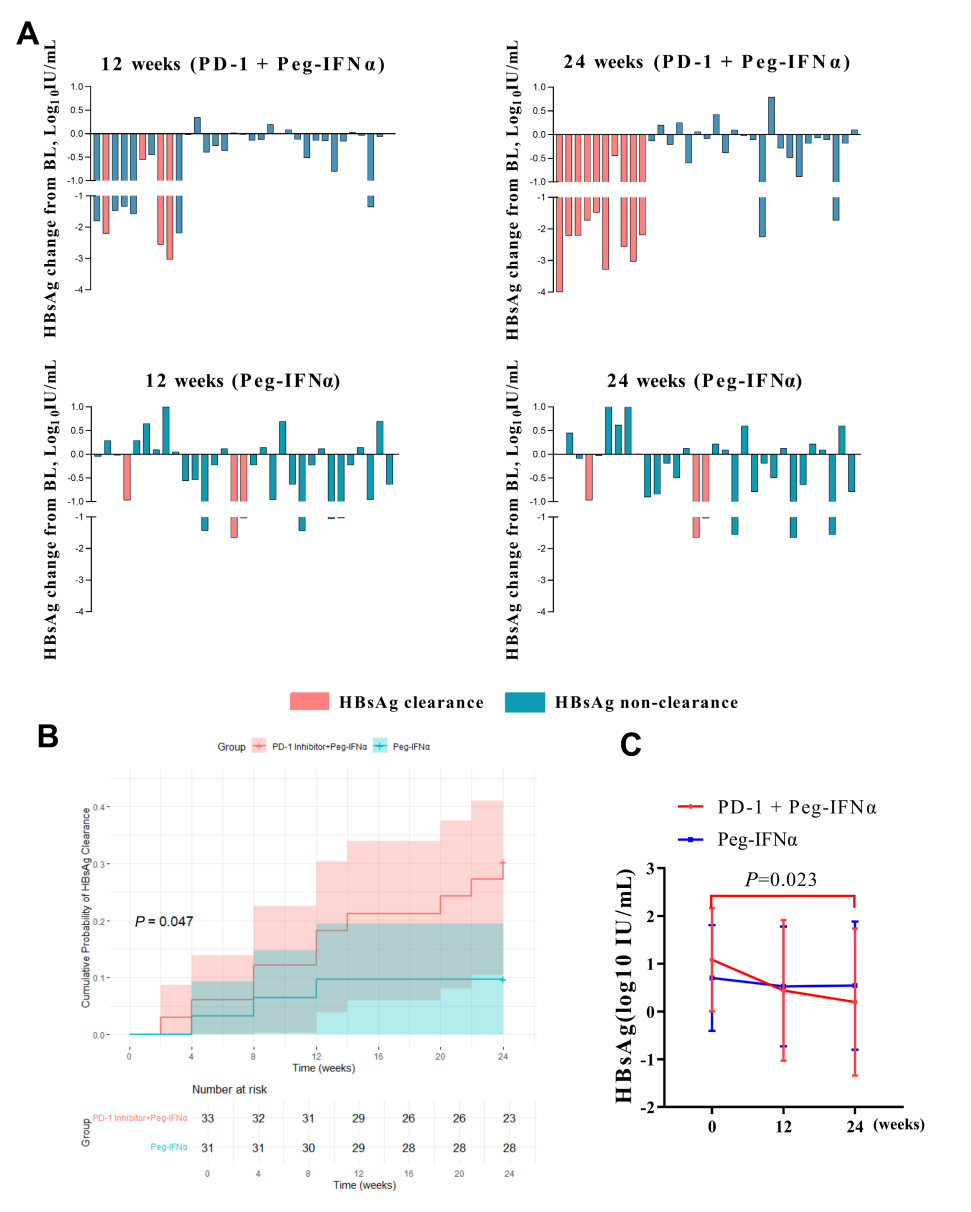
Changes in HBsAg levels from the baseline between the PD-1 inhibitor plus Peg-IFNα group and the Peg-IFNα group (A). Cumulative clearance curves of HBsAg in the PD-1 inhibitor plus Peg-IFNα group and the Peg IFN group (B). Dynamic curve of serum HBsAg in patients treated with PD-1 inhibitors or Peg-IFN (C). BL: baseline; Peg-IFNα: pegylated interferon-α.

At week 12, HBsAg loss was observed in 4/33 patients (12.1%) in the PD-1 inhibitor plus Peg-IFNα group and 2/31 patients (6.5%) in the Peg-IFNα group. By week 24, clearance rates rose to 10/33 (30.3%) and 3/31 (9.7%), respectively. The median time to HBsAg loss in the PD-1 inhibitor plus Peg-IFNα group was 12 weeks (range 2.0–20.5 weeks), with the earliest clearance occurring 2 weeks after the single PD-1 inhibitor dose. Figure 3A compares longitudinal HBsAg levels; Figure 3B depicts clearance curves. In the PD-1 inhibitor plus Peg-IFNα group, median HBsAg decreased from 0.949 (0.276–1.924) to 0.033 (-1.602–1.749) log_10_ IU/mL at week 24 ( *P* = 0.013), whereas the Peg-IFNα group showed no significant change (Figure 3C). However, HBsAg seroconversion was observed in only one case in the PD-1 inhibitor plus Peg-IFNα group (occurring at the 24-week mark), whereas no HBsAg seroconversion has been observed thus far in the control group.

### Dynamics of liver enzymes

**Figure 4:**
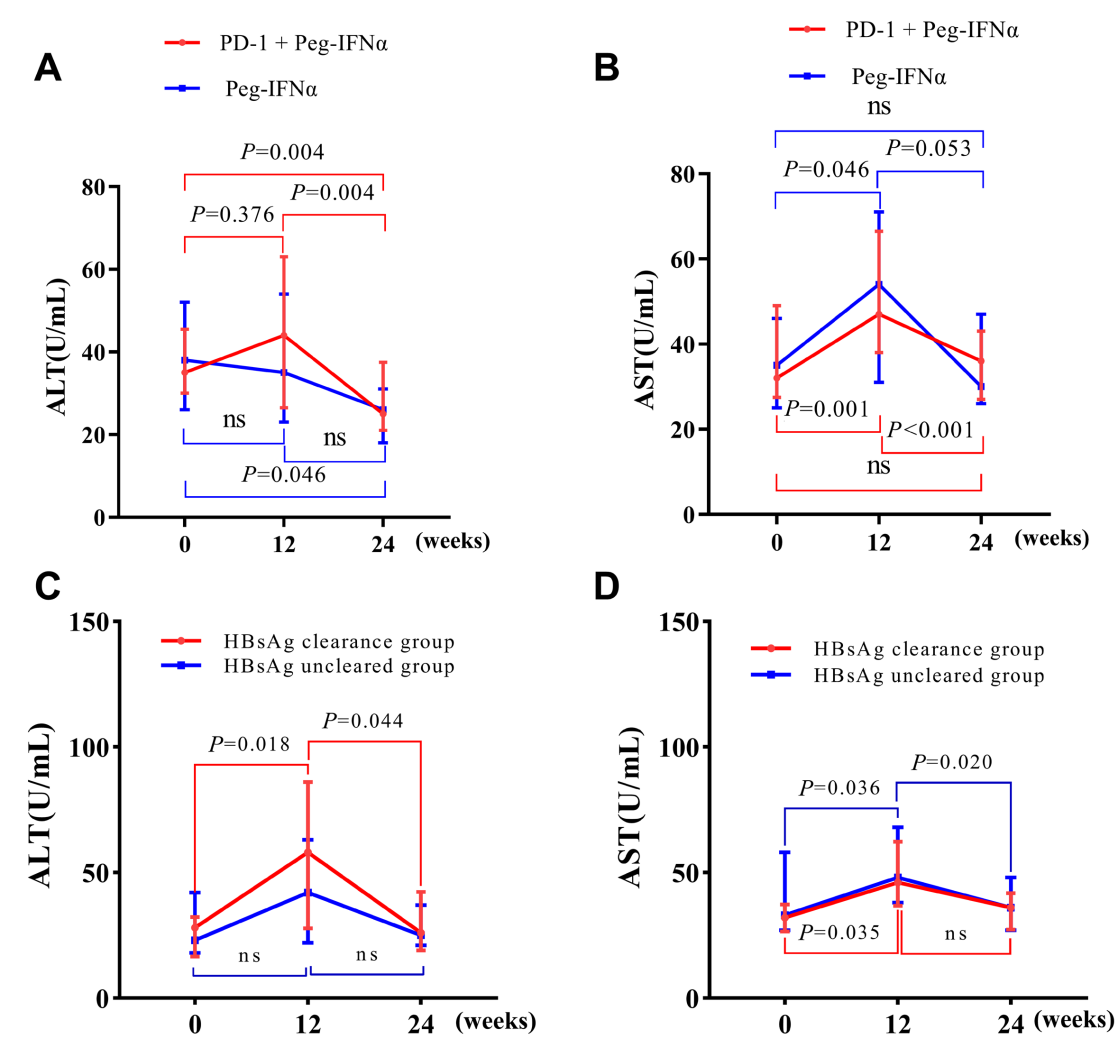
Dynamic curve of transaminase levels. Dynamic changes in the serum ALT (A) and AST (B) levels in PD-1 inhibitor plus Peg-IFNα group and Peg-IFNα group for 24 weeks. Comparison of the dynamic changes in the serum ALT (C) and AST (D) levels between patients with 24-week HBsAg clearance and patients with nonclearance of HBsAg treated with PD-1 inhibitors and add-on Peg IFN α2b. ALT: alanine transaminase; AST: aspartate transaminase. Peg-IFNα: pegylated interferon-α.

ALT and AST trajectories for both groups are presented in Figure 4. In the PD-1 inhibitor plus Peg-IFNα group, ALT and AST rose significantly at week 12, then returned to baseline by week 24 (Figure 4A and B). In the Peg-IFNα group, ALT peaked at week 24, while AST rose at week 12 and normalized by week 24. Stratifying the PD-1 inhibitor plus Peg-IFNα group cohort by HBsAg clearance revealed a pronounced ALT surge at week 12 only in patients who ultimately cleared HBsAg (Figure 4C); AST increased similarly in both subgroups (Figure 4D). These patterns suggest transient, controllable immune-mediated hepatic inflammation, with the degree of ALT elevation potentially linked to subsequent HBsAg loss.

### Safety

In the Peg-IFNα group, the observed AEs included neutropenia, thrombocytopenia, fever, fatigue, and anorexia. These AEs, whose incidences were high, were considered by the investigators to be closely related to Peg-IFN treatment. A few patients developed thyroid dysfunction, rash and alopecia during Peg-IFN treatment, and three patients had ALT flares (ALT > 5 ULN). During treatment with a PD-1 inhibitor combined with Peg-IFN, patients also experienced AEs such as neutropenia, thrombocytopenia, fever, fatigue, and anorexia. Four patients developed stomatitis during treatment, but this AE was not observed during treatment with Peg-IFN monotherapy. Additionally, during treatment with a PD-1 inhibitor, ALT flares occurred in 2 patients. The incidence showed no statistically significant difference compared with the Peg-IFN treatment group. Based on the Common Terminology Criteria for Adverse Events (CTCAE) v5.0, all these ALT flare cases were assessed as Grade 3 adverse events, and significant improvement was observed in all patients after treatment with enzyme-lowering agents such as Bicyclol Tablets.

Notably, neutropenia and thrombocytopenia are long-term AEs that are observed during Peg-IFN treatment, and these AEs may occur throughout the Peg-IFN treatment period. Fever and fatigue predominantly occur in the early stage of Peg-IFN treatment, especially during the first 12 weeks, and most AEs in these patients gradually resolve throughout long treatment periods. However, we found that during treatment with a PD-1 inhibitor combined with Peg-IFN, 12 (36.4%) patients developed fever a second time, and 15 (45.5%) patients developed fatigue a second time. Researchers believe that this may be the result of combined treatment with PD-1 inhibitors. When Peg-IFN was combined with a PD-1 inhibitor, the AEs associated with Peg-IFN monotherapy were aggravated or exacerbated. Table 2 lists the incidence of AEs observed during treatment.

**Table 2:** Adverse events in the study population, *n* (%)

AEs	Peg-IFNα group (*n* = 31)	PD-1 inhibitor plus Peg-IFNα (*n* = 33)	*P* value
Neutropenia	18 (58.1)	20 (60.6)	0.830
Thrombocytopenia	16 (51.6)	22 (66.7)	0.332
Fever	5 (16.1)	12 (36.4)	**0.043**
Fatigue	6 (19.4)	15 (45.5)	0.050
Anorexia	5 (16.1)	8 (24.2)	0.215
Thyroid dysfunction	3 (9.7)	1 (3.0)	0.561
ALT flare	3 (9.7)	2 (6.1)	0.942
Rash	2 (6.4)	3 (9.1)	1.000
Stomatitis	0 (0.0)	4 (12.1)	**0.045**
Muscle soreness	1 (3.2)	2 (6.1)	1.000
Alopecia	5 (16.1)	2 (6.1)	0.374

Bold values indicate statistically significant differences (*P* < 0.05). Peg-IFNα: pegylated interferon-α.

### Interferon-related cytokines

Nine type I IFN-associated cytokines were quantified by ELISA (IFIT1, MX1, IFI6, ISG15, OAS1, EIF2AK2, STAT1, IRF7, IFITM3). At week 24, ISG15 (45.38 ± 4.33 *vs*. 36.50 ± 6.30 ng/L; *P <* 0.001) and IRF7 (1481.9 ± 217.9 *vs*. 1113.7 ± 401.4 ng/L; *P* = 0.001) were significantly higher in the PD-1 inhibitor plus Peg-IFNα group (Table 3). Within the PD-1 inhibitor plus Peg-IFNα group, ISG15 rose steadily from baseline to week 24 (*P* = 0.028), whereas the other eight markers remained unchanged. In contrast, neither ISG15 nor IRF7 changed significantly over time in the Peg-IFNα group. When the 33 combination-treated patients were stratified by HBsAg clearance status, baseline and week-12 OAS1 levels were higher in clearers than in non-clearers (baseline 202.5 ± 22.6 *vs*. 156.8 ± 38.0 ng/L, *P* = 0.002; week 12, 204.1 ± 22.1 *vs*. 165.8 ± 48.2 ng/L, *P* = 0.034; Table 4).

**Table 3:** Interferon-related protein levels in the PD-1 inhibitor treatment and control groups

Characteristics	Peg-IFNα group (*n* = 31)	Peg-IFNα group (*n* = 31)	PD-1 inhibitor plus Peg-IFNα (*n* = 33)	*P* value^a^	*P* value^b^
	Baseline	24 weeks	24 weeks		
IFIT1	718.04±146.39	759.27±166.20	713.52±147.28	0.608	0.414
MX1	734.94±143.19	737.11±199.19	755.46±165.56	0.980	0.776
IFI6	724.89±202.31	1042.64±167.88	935.02±241.47	**0.005**	0.180
ISG15	39.75±6.20	36.50±6.30	45.38±4.33	0.327	**<0.001**
OAS1	152.76±34.72	157.64±31.48	181.49±38.22	0.782	0.075
EIF2AK2	127.66±32.24	129.53±26.63	136.83±37.58	0.907	0.557
STAT1	569.14±137.05	628.33±148.40	543.73±117.86	0.427	0.077
IRF7	1268.55±356.17	1113.70±401.35	1481.90±217.93	0.430	**0.001**
IFITM3	10.02±2.83	11.46±2.59	10.72±2.51	0.325	0.422

*P* value^a^: Peg-IFNα group (baseline) *vs*. Peg-IFNα group (24 weeks); *P* value^b^: Peg-IFNα group (24 weeks) *vs*. PD-1 inhibitor plus Peg-IFNα group (24 weeks). Bold values indicate statistically significant differences (*P* < 0.01). Peg-IFNα: pegylated interferon-α.

**Table 4:** Interferon-related protein levels in the PD-1 inhibitor treatment group

Characteristics	PD-1 inhibitor plus Peg-IFNa group (*n* = 33)	PD-1 inhibitor plus Peg-IFNa group (*n* = 33)	PD-1 inhibitor plus Peg-IFNa group (*n* = 33)	*P* value	HBsAg clearance Group (*n* = 10)	HBsAg nonclearance Group (*n* = 23)	*P* value	HBsAg clearance Group (*n* = 10)	HBsAg nonclearance Group (*n* = 23)	*P* value
	Baseline	12 weeks	24 weeks		Baseline	Baseline		12 weeks	12 weeks	
IFIT1	723.13 ± 168.42	720.28 ± 187.39	713.52 ± 147.28	0.422	737.66 ± 198.21	721.2 ± 173.54	0.815	733.02 ± 257.31	692.14 ± 181.19	0.640
MX1	656.07 ± 184.97	647.31 ± 187.96	755.46 ± 165.56	0.340	638.33 ± 191.43	671.22 ± 188.2	0.654	656.97 ± 243.60	669.79 ± 183.81	0.881
IFI6	903.77 ± 247.22	969.58 ± 263.56	935.02 ± 241.47	0.698	1011.52 ± 250.37	877.51 ± 258.9	0.184	1070.56 ± 305.76	898.96 ± 284.72	0.167
ISG15	36.73 ± 7.99	40.67 ± 7.71	45.38 ± 4.33	0.028	38.95 ± 7.84	37.34 ± 7.93	0.600	41.88 + 8.36	37.98 ± 7.57	0.238
OAS1	172.23 ± 38.78	170.54 ± 46.1	181.49 ± 38.22	0.637	202.54 ± 22.63	156.78 ± 38.03	**0.002**	204.14 ± 22.09	165.78 ± 48.23	**0.034**
EIF2AK2	126.49 ± 36.55	130.06 ± 38.44	136.83 ± 37.58	0.846	143.00 ± 34.33	121.29 ± 31.99	0.095	146.08 ± 44.36	130.32 ± 32.38	0.309
STAT1	568.31 ± 126.82	580.29 ± 137.87	543.73 ± 117.86	0.609	620.36 ± 134.82	554.94 ± 121.96	0.187	586.58 ± 152.42	567.48 ± 129.85	0.740
IRF7	1262.3 ± 350.77	1368.65 ± 317.9	1481.90 ± 217.93	0.260	1185.91 ± 348.35	1357.47 ± 298.10	0.166	1222.29 ± 269.44	1460.92 ± 295.41	0.055
IFITM3	11.38 ± 3.14	11.81 ± 3.07	10.72 ± 2.51	0.747	11.16 ± 3.44	11.99 ± 3.22	0.515	11.87 ± 3.10	11.54 ± 3.09	0.800

Bold values indicate statistically significant differences (*P* < 0.05). Peg-IFNα: pegylated interferon-α.

**Figure 5:**
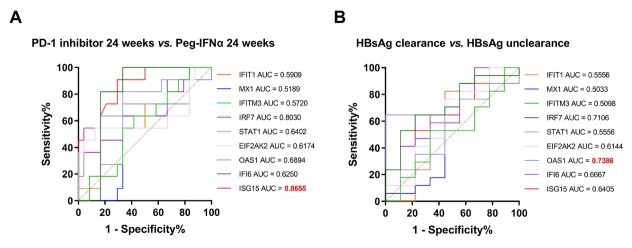
ROC curves of nine interferon-related cytokines. ROC curves of interferon-related cytokines at 24 weeks in the PD-1 inhibitors plus Peg-IFN group *vs*. the Peg-IFN group (A); ROC curves of interferon-related cytokines in patients with vs. without HBsAg clearance in the PD-1 inhibitors plus Peg-IFN group (B). ROC: receiver operating characteristic; Peg-IFN: pegylated interferon.

To further validate their predictive value, receiver operating characteristic (ROC) analyses were performed. At week 24, ISG15 yielded an area under the curve (AUC) of 0.866 (95% CI: 0.765–0.966, *P* < 0.001) for distinguishing the PD-1 inhibitor plus Peg-IFNα group from the Peg-IFNα monotherapy group (Figure 5A). Within the PD-1 inhibitor plus Peg-IFNα group, OAS1 achieved an AUC of 0.739 (95% CI: 0.546–0.931, *P* = 0.049) for differentiating patients with HBsAg clearance from those without clearance (Figure 5B).

These findings implicate elevated OAS1—and sustained ISG15 induction—as potential biomarkers of favorable response to PD-1 inhibition plus Peg-IFNα, underscoring interplay between PD-1 blockade and the interferon pathway.

## Discussion

PD-1 inhibitors have demonstrated favorable safety and efficacy profiles in hepatocellular carcinoma (HCC), with increasing interest in their application for CHB.^[17]^ Retrospective studies have shown that PD-1/PD-L1 blockade may facilitate HBsAg clearance in HCC patients undergoing immunotherapy.^[18]^ In 2019, Gane *et al*. reported the first administration of the PD-1 inhibitor nivolumab in non-HCC CHB patients, showing good tolerability at a low dose of 0.3 mg/kg.^[19]^ Accumulating evidence suggests that long-term Peg-IFNα treatment may impair T cell responses by upregulating PD-1, contributing to suboptimal HBsAg clearance.^[14]^ Meanwhile, previous studies in HCC have suggested that Peg-IFNα may enhance the efficacy of PD-1 inhibitors by augmenting T cell priming and activation.^[20,21]^ In our study, we investigated the addition of the PD-1 inhibitor sintilimab to Peg-IFNα in 33 CHB patients who failed to clear HBsAg after 48 weeks of Peg-IFNα therapy and exhibited elevated PD-1 expression in peripheral blood T cells. A comparator group of 31 patients continued Peg-IFNα therapy. Notably, HBsAg clearance occurred as early as 2 weeks post–PD-1 inhibitor administration. At 24 weeks, 30.3% (10/33) of patients in the combination group achieved HBsAg clearance, significantly higher than the 9.7% (3/31) clearance rate observed in the Peg-IFNα group. These findings suggest that PD-1 inhibitors may act synergistically with Peg-IFNα to restore antiviral immunity and promote HBsAg clearance.

Currently, there are limited treatment strategies for CHB patients who do not achieve HBsAg clearance after 48 weeks of Peg-IFNα therapy. The prolonged interferon therapy in the New Switch study reported HBsAg clearance rates of 14.4% and 20.7% at 48 and 96 weeks in patients with undetectable HBV DNA and HBeAg negativity.^[22]^ Indicating that prolonged treatment may increase the rate of HBsAg clearance. Intermittent Peg-IFNα therapy has demonstrated a 19.4% clearance rate in patients experiencing a plateau of HBsAg decline after 48 weeks of Peg-IFNα treatment.^[23]^ Our findings provide a potentially more effective option for this difficult-to-treat population, particularly in those with elevated PD-1 expression. To our knowledge, this is the first clinical study to evaluate PD-1 blockade combined with Peg-IFNα in CHB, and our findings provide important proof-of-concept data for this combination strategy. Meanwhile, transient increases in ALT were observed in the Peg-IFNα plus PD-1 inhibitor group, peaking at 12 weeks and normalizing by week 24. Importantly, patients who achieved HBsAg clearance showed significantly higher ALT levels at 12 weeks than non-responders. These ALT flares may reflect immune reactivation and could serve as response biomarkers.

Regarding safety, the combination regimen was well tolerated, even at a sintilimab dose of 1 mg/ kg. The majority of AEs—including neutropenia, thrombocytopenia, fatigue, and anorexia—were consistent with known Peg-IFNα effects. However, reactivation of typical Peg-IFNα–associated AEs, such as fever and fatigue, was more frequent in the combination group.^[24]^ These findings suggest that PD-1 blockade may enhance not only the therapeutic effects but also the immune-related toxicity of Peg-IFNα, necessitating close clinical monitoring. Stomatitis, observed in 12.1% of patients in the combination group and not in the Peg-IFNα group, may be attributable to the PD-1 inhibitor, although a synergistic effect cannot be ruled out.

Some scholars have proposed that the addition of Peg-IFNα can increase the efficacy of a PD-1 inhibitor in the treatment of HCC.^[20,21]^ The treatment effect, to some extent, explains the interaction between Peg-IFNα and PD-1 inhibitors. In patients with CHB, the addition of a PD-1 inhibitor may also strengthen their response to Peg-IFNα treatment, thereby promoting HBsAg clearance. However, PD-1 inhibitors may not only strengthen the therapeutic effect of Peg-IFNα, but they may also exacerbate or evoke the original AEs caused by Peg-IFNα; therefore, clinicians should pay special attention to these dual effects of PD-1 inhibitors in clinical practice.

Mechanistically, the combination appeared to modulate the interferon signaling cascade. Type I interferons act *via* Janus kinase/signal transducer and activator of transcription (JAK/STAT) pathway and induce interferon-stimulated genes (ISGs)(*e.g*., ISG15, MX1, OAS1, IFITM3). Using ELISA, we assessed serum levels of nine ISG-related proteins and found that ISG15 and IRF7 levels were significantly elevated in the Peg-IFNα plus PD-1 inhibitor group at 24 weeks compared to Peg-IFNα group. ISG15 trended upward during treatment in the combination group, but the same change was not observed in IRF7, and neither ISG15 nor IRF7 changed significantly in the Peg-IFNα group, suggesting PD-1 inhibitors potentiate interferon signaling *via* ISG15 induction.

Stratifying by response in the Peg-IFNα plus PD-1 group, HBsAg-clearing patients had higher OAS1 levels at baseline and 12 weeks. These findings indicate that high levels of OAS1 may contribute to HBsAg clearance. This is consistent with recent transcriptomic data from CHB liver biopsies showing stronger ISG induction, including OAS1, in patients who responded to interferon therapy.^[25]^ Interestingly, responders in that study exhibited lower baseline ISG expression, suggesting preserved interferon responsiveness as a determinant of treatment success. Our data extend this observation and suggest that OAS1 could serve as a predictive biomarker for interferon-PD-1 combination therapy.

This study has several limitations. Firstly, the sample size was modest and may limit generalizability. Secondly, the PD-1 inhibitor dose (1 mg/ kg) was selected based on prior exploratory studies and may not represent the optimal therapeutic concentration. Thirdly, the nonrandomized design, driven by patient preference, carries inherent selection bias, limiting the robustness of causal conclusions. Finally, we assessed 24-week HBsAg clearance as the primary endpoint in patients who had not achieved clearance after 48 weeks of Peg-IFNα therapy. Although this endpoint provides valuable information on the short-term antiviral and immunological effects of PD-1–based combination therapy, longer-term follow-up is required to determine the durability of HBsAg clearance and to evaluate its potential to contribute to functional cure. Future work should include larger, multicenter, randomized controlled cohorts and employ dose-escalation strategies to optimize the risk–benefit profile of PD-1–based combination immunotherapy in CHB. Furthermore, we will continue follow-up to supplement data on sustained HBsAg loss.

In summary, our findings suggest that combining PD-1 inhibitors with Peg-IFNα can significantly increase HBsAg clearance in CHB patients with prior Peg-IFNα failure and elevated PD-1 expression. The therapy was generally well tolerated and elicited meaningful immune activation, as evidenced by ALT flares and ISG upregulation. Importantly, OAS1 emerged as a potential biomarker of response. These preliminary findings provide a rationale for further validation in larger, multicenter randomized controlled trials.

## Supplementary Material

Supplementary Material Details

## References

[1] Terrault NA, Bzowej NH, Chang K-M, Hwang JP, Jonas MM, Murad MH (2016). AASLD guidelines for treatment of chronic hepatitis B.. Hepatology.

[2] Mak L-Y, Cheung K-S, Hui RW, Wong DK, Fung J, Yuen M-F (2022). Enhanced liver fibrosis score stratifies hepatocellular carcinoma risk in patients with hepatitis B surface antigen seroclearance. Clin Infect Dis.

[3] Choi W-M, Yip TC, Wong GL, Kim WR, Yee LJ, Brooks-Rooney C (2025). Baseline viral load and on-treatment hepatocellular carcinoma risk in chronic hepatitis B: a multinational cohort study. Clin Gastroenterol Hepatol.

[4] Zheng H, Wang Y, Wang F, Shen L, Zhang G, Liu J (2024). New progress in HBV control and the cascade of health care for people living with HBV in China: evidence from the fourth national serological survey, 2020. Lancet Reg Health West Pac.

[5] Yan R, Sun M, Yang H, Du S, Sun L, Mao Y. (2024). latest report on hepatitis B virus epidemiology in China: current status, changing trajectory, and challenges. Hepatobiliary Surg Nutr 2025.

[6] Wang M, Wang Y, Feng X, Wang R, Wang Y, Zeng H (2017). Contribution of hepatitis B virus and hepatitis C virus to liver cancer in China north areas: Experience of the Chinese National Cancer Center. Int J Infect Dis.

[7] Zhang M, Wan M, Wang W, Lin S, Zhang X (2024). Effect of interferon therapy on quality of life in patients with chronic hepatitis B.. Sci Rep.

[8] Ambler R, Edmunds GL, Tan SL, Cirillo S, Pernes JI, Ruan X (2020). PD-1 suppresses the maintenance of cell couples between cytotoxic T cells and target tumor cells within the tumor. Sci Signal.

[9] Paillon N, Mouro V, Dogniaux S, Maurin M, Saez Pons J-J, Ferran H (2023). PD-1 inhibits T cell actin remodeling at the immunological synapse independently of its signaling motifs. Sci Signal.

[10] Attanasio J, Wherry EJ (2016). Costimulatory and coinhibitory receptor pathways in infectious disease. Immunity.

[11] Tinoco R, Carrette F, Barraza ML, Otero DC, Magaña J, Bosenberg MW (2016). PSGL-1 is an immune checkpoint regulator that promotes T cell exhaustion. Immunity.

[12] Su M, Ye T, Wu W, Shu Z, Xia Q (2024). Possibility of PD-1/PD-L1 inhibitors for the treatment of patients with chronic hepatitis B infection. Dig Dis.

[13] Féray C, López-Labrador FX (2019). Is PD-1 blockade a potential therapy for HBV JHEP Rep..

[14] Zhu Y, Chen M, Xu D, Li TE, Zhang Z, Li JH (2022). The combination of PD-1 blockade with interferon-α has a synergistic effect on hepatocellular carcinoma. Cell Mol Immunol.

[15] Meng CY, Sun S, Liang Y, Xu H, Zhang C, Zhang M (2023). Engineered anti-PDL1 with IFNα targets both immunoinhibitory and activating signals in the liver to break HBV immune tolerance. Gut.

[16] Wang J, Fei K, Jing H, Wu Z, Wu W, Zhou S (2019). Durable blockade of PD-1 signaling links preclinical efficacy of sintilimab to its clinical benefit. MAbs.

[17] Galle PR, Forner A, Llovet JM, Mazzaferro V, Piscaglia F, Raoul J-L (2018). EASL Clinical Practice Guidelines: Management of hepatocellular carcinoma. J Hepatol.

[18] Mon H-C, Lee P-C, Hung Y-P, Hung Y-W, Wu C-J, Lee C-J (2025). Functional cure of hepatitis B in patients with cancer undergoing immune checkpoint inhibitor therapy. J Hepatol.

[19] Gane E, Verdon DJ, Brooks AE, Gaggar A, Nguyen AH, Subramanian GM (2019). Anti-PD-1 blockade with nivolumab with and without therapeutic vaccination for virally suppressed chronic hepatitis B: A pilot study. J Hepatol.

[20] Hu B, Yu M, Ma X, Sun J, Liu C, Wang C (2022). IFNα potentiates anti-PD-1 efficacy by remodeling glucose metabolism in the hepatocellular carcinoma microenvironment. Cancer Discov.

[21] Kao K-C, Jaccard A, Ho P-C (2022). IFNα potentiates immune-checkpoint blockade by rewiring metabolic cross-talk. Cancer Discov.

[22] Hu P, Shang J, Zhang W, Gong G, Li Y, Chen X (2018). HBsAg loss with peg-interferon Alfa-2a in hepatitis B patients with partial response to nucleos(t)ide analog: new switch study. J Clin Transl Hepatol.

[23] Li M, Xie S, Bi X, Sun F, Zeng Z, Deng W (2022). An optimized mode of interferon intermittent therapy help improve HBsAg disappearance in chronic hepatitis B patients. Front Microbiol.

[24] Wu FP, Yang Y, Li M, Liu YX, Li YP, Wang WJ (2020). Add-on pegylated interferon augments hepatitis B surface antigen clearance vs continuous nucleos(t)ide analog monotherapy in Chinese patients with chronic hepatitis B and hepatitis B surface antigen≤1500 IU/mL: an observational study. World J Gastroenterol.

[25] Li N, Yu K, Dong M, Wang J, Yang F, Zhu H (2022). Intrahepatic transcriptomics reveals gene signatures in chronic hepatitis B patients responded to interferon therapy. Emerg Microbes Infect.

