# Supplementary material for: Add-on PD-1 inhibitor with Peg-IFNα therapy favors functional cure of chronic hepatitis B patients
Source: J Transl Int Med. 2026 Feb 13;14(1):123–33. doi: 10.1515/jtim-2026-0018 (PMC12916265; doi:10.1515/jtim-2026-0018)
Supplement: Supplementary file 1 — Supplementary Material Details [file jtim-2026-0018_sm.pdf]

Supplementary materials

Supplementary Table S1: Dilution Concentration of Standard Samples in ELISA Experiments

Cytokine	Standard dilution concentration gradient (S0-S5)
OAS1	0, 15, 30, 60, 120, 240 pg/mL
IFI6	0, 75, 150, 300, 600, 1200 pg/mL
IRF7	0, 100, 200, 400, 800, 1600 pg/mL
ISG15	0, 3, 6, 12, 24, 48 ng/mL
IFITM3	0, 1, 2, 4, 8, 16 ng/mL
MX1	0, 62.5, 125, 250, 500, 1000 pg/mL
IFIT1	0, 62.5, 125, 250, 500, 1000 pg/mL
EIF2AK2	0, 12.5, 25, 50, 100, 200 pg/mL
STAT1	0, 50, 100, 200, 400, 800 pg/mL
